# The Bacterial Flagellar Motor: Insights Into Torque Generation, Rotational Switching, and Mechanosensing

**DOI:** 10.3389/fmicb.2022.911114

**Published:** 2022-05-30

**Authors:** Shuaiqi Guo, Jun Liu

**Affiliations:** ^1^Microbial Sciences Institute, Yale University, West Haven, CT, United States; ^2^Department of Microbial Pathogenesis, Yale School of Medicine, New Haven, CT, United States

**Keywords:** rotary motor, molecular machine, rotational switching, torque generation, electron tomography

## Abstract

The flagellar motor is a bidirectional rotary nanomachine used by many bacteria to sense and move through environments of varying complexity. The bidirectional rotation of the motor is governed by interactions between the inner membrane-associated stator units and the C-ring in the cytoplasm. In this review, we take a structural biology perspective to discuss the distinct conformations of the stator complex and the C-ring that regulate bacterial motility by switching rotational direction between the clockwise (CW) and counterclockwise (CCW) senses. We further contextualize recent *in situ* structural insights into the modulation of the stator units by accessory proteins, such as FliL, to generate full torque. The dynamic structural remodeling of the C-ring and stator complexes as well as their association with signaling and accessory molecules provide a mechanistic basis for how bacteria adjust motility to sense, move through, and survive in specific niches both outside and within host cells and tissues.

## The Flagellar Motor Propels Bacterial Motility in Various Environments

More than 300 years after Antonie van Leeuwenhoek discovered minuscule moving creatures using his homemade single-lensed microscope, we are now beginning to unravel the molecular details that govern bacterial motility. Bacteria have evolved distinct mechanisms to adapt to and navigate through environments of varying complexity (Wadhwa and Berg, 2021). The flagellum is a molecular nanomachine used by many bacteria for swimming motility (Berg and Anderson, 1973; Berg, 2003; Nakamura and Minamino, 2019) and for responding to environmental cues, such as chemotaxis to move to favorable locations, sense mechanical stimuli (i.e., surfaces), and initiate colonization of a niche by forming surface-attached communities known as biofilms (Lele et al., 2013; Belas, 2014; Hug et al., 2017; Nord et al., 2017; Gordon and Wang, 2019). In this review, we summarize recent findings that address longstanding fundamental questions about torque generation and rotational switching by the flagellar motor. In addition, we discuss new findings that demonstrate how periplasmic accessory proteins, such as the transmembrane protein FliL with previously unclear functions, enhance flagellar motor performance. Overall, this review aims to provide mechanistic insights into how diverse bacteria sense environments and adjust motility *via* their rotary flagellar motors.

## The Flagellar Motor is a Dynamic Supramolecular Nanomachine

**Figure 1:**
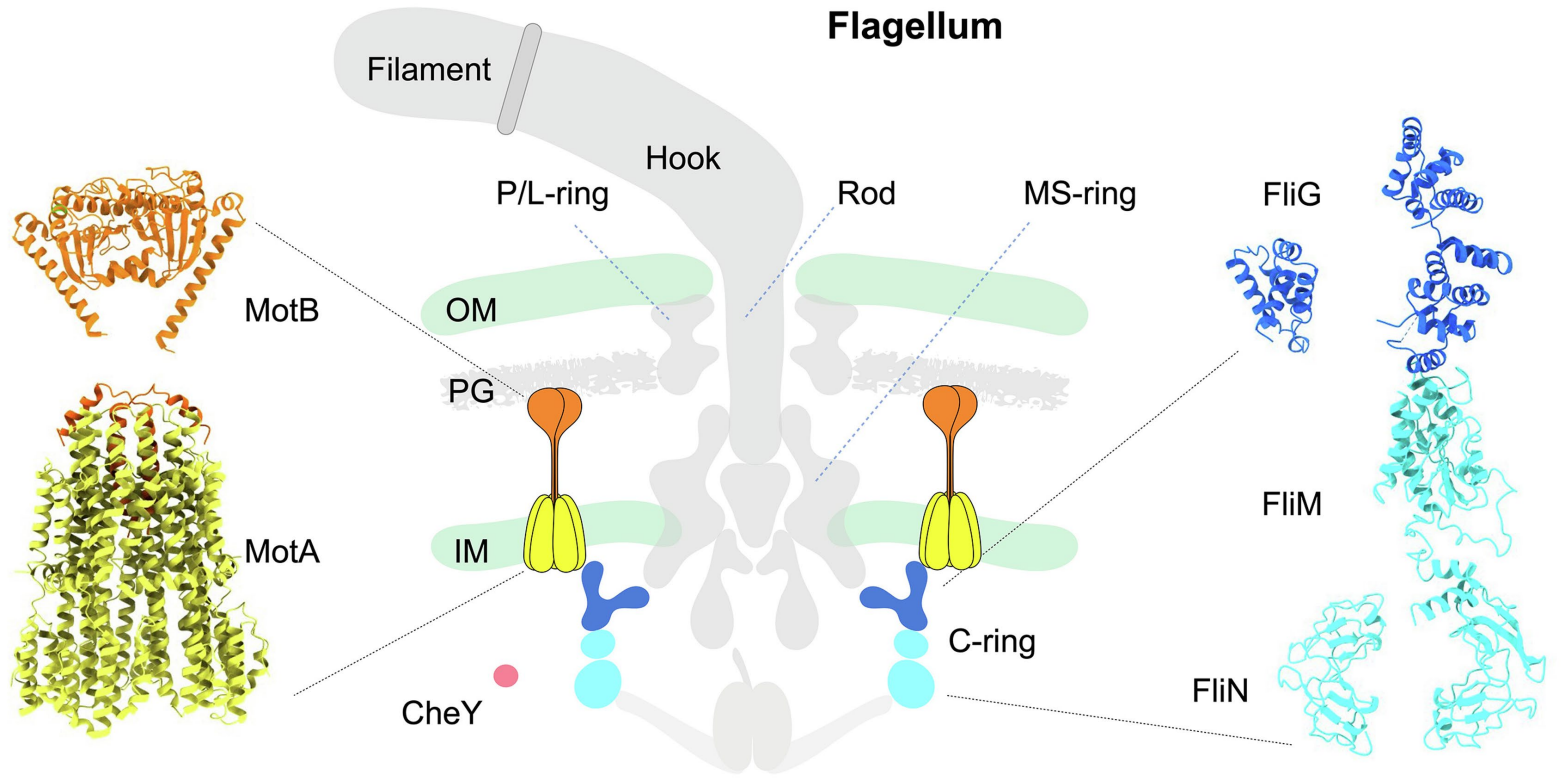
Schematic diagram illustrating the architecture of the canonical flagellum from *Escherichia coli*. Middle panel: The flagellum is composed of the extracellular filament, a flexible hook, and a motor spanning the cell envelope. The motor is further composed of a central rotor surrounded by multiple stator units. The MS-ring is anchored in the inner membrane. Above the MS-ring are the P- and L-rings associated with the PG and outer membrane. Beneath the MS-ring is the C-ring (blue and cyan) in the cytoplasm. Each stator complex has two subunits: the pentameric cone-shaped MotA (yellow) is embedded in the inner membrane, and the extended MotB (orange) is anchored to the PG on one end, while its other end is inserted into MotA. CheY-like chemotactic signaling proteins are illustrated as a red sphere. OM: outer membrane. IM: inner membrane. PG: peptidoglycan. Left panel: Structure of a stator complex in its compact, inactive conformation. The structure of MotA (yellow) in complex with partial MotB (N-terminal helix, orange) from *Campylobacter jejuni* was resolved by cryo-EM single-particle analysis (PDB: 6ykm). The MotB_PGB_ structure (C-terminal domain of MotB; orange) from *Salmonella enterica* was determined by X-ray crystallography (PDB: 2zvz). Right panel: Pseudoatomic structure of a C-ring subunit rotating in the CCW direction. The FliG subunit is colored blue, and the FliM/N subunits are colored cyan. The molecular models of individual C-ring proteins FliG, FliM, and FliN were generated with ITASSER using respective X-ray crystal structures as templates (PDBs of FliG domains: 4FHR, 3HJL; PDBs of FliM: 4FHR, 4YXB; PDB for FliN: 1YAB). Assembly of these proteins was guided by the C-ring architecture resolved by cryo-ET and subtomogram averaging.

A canonical flagellum, such as that produced by *Escherichia coli*, is composed of an extracellular filament, a cell envelope-spanning rotary motor, and a flexible hook that joins the filament with the motor (Berg, 2003; Nakamura and Minamino, 2019; Figure 1 middle panel). The flagellar motor, which powers the rotation of the filament, is composed of approximately 25 different proteins that include a central rotor surrounded by multiple inner membrane-associated stator complexes (Figure 1 left panel). Within the rotor, the shaft-like rod engages with the MS-ring embedded in the inner membrane. The C-ring, also known as the switch complex, is a larger structure attached to the MS-ring from the cytoplasm (Figure 1 middle and right panels), and its interaction with the stator complexes is critical for the rotation and directional switching of the flagellar motor. The L- and P-rings are fixed to the outer membrane and peptidoglycan, respectively, which act as bushings to support the rotor to transmit torque to the filament *via* the flexible hook in the extracellular environment (Yamaguchi et al., 2021).

Despite intensive research over the past several decades, there has been a lack of mechanistic understanding of how the stator complexes activate the rotation of the rotor. In the past few years, technical breakthroughs in imaging tools, particularly cryo-electron microscopy (cryo-EM) and cryo-electron tomography (cryo-ET), have revealed unprecedented structural details of the individual motor components that make up the torque generation device as well as insights into how they cohesively work together as a dynamic nanomachine.

## Stator Complexes are the Torque Generators That Drive C-Ring Rotation

**Figure 2:**
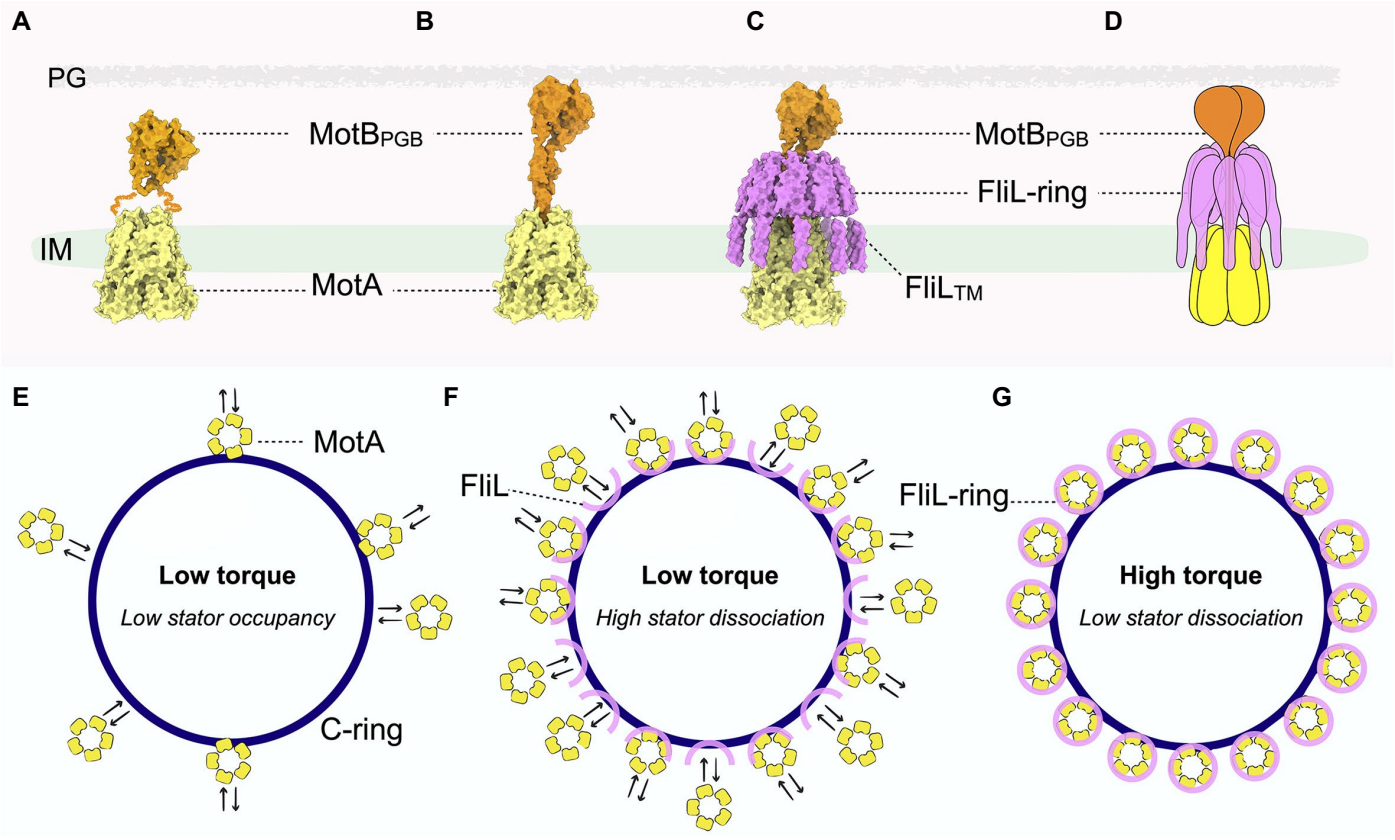
Pseudoatomic structure of the stator-FliL-supramolecular complex and proposed model for modulating torque generation. **(A)** Side view of the stator structure in its compact, inactive conformation. MotA is colored yellow and MotB orange. The squiggly orange lines indicate the flexible MotB linkers with unknown structure. The cryo-ET map for the inner membrane is colored green. **(B)** Side view of the pseudoatomic model of the stator complex in its extended, active conformation. **(C)** Side view of the pseudoatomic model of the stator-FliL supramolecular complex. The FliL-ring is colored magenta. **(D)** Cartoon representation of the stator-FliL supramolecular complex. **(E)** In the absence of FliL, the stator units rapidly bind and unbind the rotor. Low stator occupancy results in low torque production by the motor. **(F)** Under low-load conditions, stator units bind and unbind the rotor rapidly before FliL can properly assemble into full rings to enclose the stator. **(G)** Under high-load conditions, FliL forms a full ring to surround and stabilize the stator complex in its active, extended conformation to maximize PMF to generate high torque.

The rotation of the rotor requires the presence of one or more torque-generating stator units (Wadhwa and Berg, 2021). Each stator complex is composed of two proteins: MotA and MotB (Tang et al., 1996). MotA is an inner membrane-associated protein with a large cytoplasmic domain that directly interacts with the rotor (Dean et al., 1984; Blair and Berg, 1991; Zhou et al., 1995). MotB is primarily situated in the periplasmic space, except for its N-terminal domain, which acts as a plug inserted into the interior of MotA in the inner membrane (Kojima and Blair, 2001; Hosking et al., 2006; Roujeinikova, 2008; Kojima et al., 2009, 2018; Yonekura et al., 2011; Chang et al., 2021; Homma et al., 2021; Terashima et al., 2021; Figure 1 left panel). At its C terminus, MotB contains a peptidoglycan-binding domain (MotB_PGB_) critical for activation of the stator ion channel (Figure 2A). While the diffusive stator unit engages with the C-ring, MotB undergoes a large conformational change, whereby MotB_PGB_ moves upward to anchor the peptidoglycan, thus unplugging the MotA ion channel (Figures 2A,B). As H^+^ or Na^+^ ions flow through the stator channel, electrochemical potential across the membrane powers rotation of the C-ring (Sharp et al., 1995a,b; Tang et al., 1996; Sato and Homma, 2000; Braun and Blair, 2001; Hosking et al., 2006; Takekawa et al., 2020). Because all components of the rotor rotate as a single unit, torque is transmitted from the C-ring to propel the rotation of the flagellar filament on the outside of the cell, thus achieving flagella-based motility.

Several high-resolution crystal structures of MotB_PGB_ (called PomB_PGB_ in *Vibrio alginolyticus*) are available, revealing that MotB_PGB_ forms a dimer critical for binding peptidoglycan and activating the stator unit (Roujeinikova, 2008; Kojima et al., 2009; Zhu et al., 2014). Until recently, structural details of MotA as well as the N-terminal region of MotB were unknown. For almost a decade, functional mutagenesis and molecular dynamics studies of the stator complex relied on a low-resolution map (~20 Å) determined by single-particle analysis with negative staining (Yonekura et al., 2011). Although the structure revealed an overall compact shape of the stator complex in its inactive conformation, the ambiguity of the low-resolution map as well as biochemical results led to the interpretation that the stator complex is comprised of two MotB subunits in complex with four MotA subunits (Kojima and Blair, 2004; Yonekura et al., 2011; Takekawa et al., 2016). This lack of well-resolved structural details of the stator complex hampered efforts to elucidate the mechanism of torque generation.

In 2020, several near-atomic resolution structures of partial stator complexes were resolved independently by two groups using cryo-EM single-particle analysis (Deme et al., 2020; Santiveri et al., 2020; Hu et al., 2021; Figures 1 left panel, 2A). These structures revealed, unexpectedly, that MotA forms a pentameric (not tetrameric), hollow cone-shaped structure that peripherally surrounds a MotB dimer. With high-resolution structural features clearly resolved, these remarkable studies indicate that protons in the periplasm are shuttled throughout key charged residues between MotA and MotB to the cytoplasm. It was proposed that the flux of ions across the inner membrane triggers 36^o^ stepwise rotations of the MotA pentamer relative to the immobile MotB dimer (Santiveri et al., 2020), providing a mechanistic model in which torque generation requires the gyration of the stator complexes.

Also in 2020, two cryo-ET studies revealed that the stator complexes and the C-ring interact intimately with each other *in situ* (Carroll et al., 2020; Chang et al., 2020, 2021). Viewed from the top, the multiple stator complexes that surround and interact with the C-ring resemble how cogwheels engage and drive the rotation of a conveyor belt (Figures 2E–G). Consistent with the described model based on near-atomic stator structures, it was further proposed that ion motive force triggers the stator units to rotate, which then drives rotation of the C-ring in a cogwheel-like manner. This elegant model, based on a combination of *in vitro* cryo-EM and *in situ* cryo-ET data, provides critical mechanistic insights that significantly shift how the field views the longstanding question: how do the stator complexes couple ion motive force to generate torque? The model suggests that the stator complexes act as an array of rotary motors themselves to drive rotation of the rotor.

## The Enigmatic Flagellar Protein FliL Likely Modulates Stator Function in Many Bacteria

With the prevailing view now shifted to regard stator complexes as rotary motors themselves, it is essential to understand how these dynamic structures are stabilized around the rotor. Though the cryo-EM single-particle studies described above aimed at solving high-resolution structures of the entire stator complex (Deme et al., 2020; Santiveri et al., 2020), MotB was only partially visible in these structures (Figure 2A). In the inactive stator complex, the N-terminal MotB dimeric domains are in *trans* conformation, wedged above MotA to plug the ion channel (Figures 1 left panel, 2A). By contrast, mutations that lock MotB in an active conformation revealed that the plug lifts to help open the MotA channel. However, the C-terminal MotB_PGB_ as well as the flexible linker region that connects MotB_PGB_ to the MotB N-terminal region were not visible in these cryo-EM structures of the stator complex (Figure 2A). Although this is likely due to a high degree of flexibility of MotB, it is also possible that the flexible linkers of MotB were proteolyzed during the purification steps. These observations raise key questions about the mechanism by which the stator complexes stabilize in their active and extend conformations *in situ*. How is the dynamic MotA stabilized and how do the flexible MotB linkers withstand the large torsional force during rapid rotation of MotA without compromising stator function and thus negatively impacting motility?

FliL is a 20-kDa inner membrane-associated protein produced by all flagellated bacteria (Jenal et al., 1994; Raha et al., 1994; Belas and Suvanasuthi, 2005; Attmannspacher et al., 2008). The *fliL* gene is often in the first position of the operon that also transcribes the C-ring genes *fliM* and *fliN*, or it is adjacent to stator genes *motA* and *motB* within the same operon (Partridge et al., 2015), suggesting that FliL interacts with the key components of the torque-generating complexes. However, since the discovery of FliL over 30 years ago, its role in torque generation has remained under debate. In fact, FliL has until now been described as a protein with “enigmatic” or “mysterious” functions, given its apparently different roles in diverse bacteria (Cusick et al., 2012).

An early study by Jenal et al. demonstrated in 1994 that FliL in *Caulobacter crescentus* is essential for flagellar rotation and bacterial motility (Jenal et al., 1994). By contrast, another study in the same year indicated that loss of FliL appeared to have a minimal effect on the swimming motility of *E. coli*, suggesting that it is not part of the flagellar nanomachinery (Raha et al., 1994). Consistent with the earlier results in *E. coli*, more recent studies have demonstrated that loss of FliL causes only small defects in swimming motility in *E. coli* and *Salmonella* (Attmannspacher et al., 2008; Partridge et al., 2015). Intriguingly, it has emerged that FliL is required for the swarming motility of *E. coli* and *Salmonella* when they encounter environments with higher external load, such as semi-solid agar media. Closer examination of Δ*fliL* mutants revealed that flagellar filaments more easily break off from bacteria under swarming conditions, suggesting a role for FliL in stabilizing the filament to the flagellar basal body. In addition, *E. coli* and *Salmonella* flagella without FliL appear to switch rotational direction less frequently. Although FliL had been implicated in the surface(mechano)-sensing pathway of the urinary tract pathogen *Proteus mirabilis* (Belas and Suvanasuthi, 2005), more recently, Chawla et al. (2017) demonstrated that FliL is not involved in mechanosensing by *E. coli*.

In the Lyme disease-causing spirochete *Borrelia burgdorferi*, loss of FliL causes noticeable defects in motility (Motaleb et al., 2011). Cryo-ET studies indicated that periplasmic flagellar filaments of Δ*fliL* mutant *B. burgdorferi* have abnormal orientations compared to those of wild type, with stator occupancy reduced to approximately 40% (Chang et al., 2019). As a mechanism to counter resistive forces in high-load environments, more stator complexes are typically recruited to the flagellar motor to produce higher torque (Figures 2E–G). A significantly lower stator occupancy in the absence of FliL again implicated this protein in torque generation *via* mediation of stator assembly to the rotor. Although cryo-ET and subtomogram averaging further indicated that FliL is localized between the rotor and stator complex in the periplasmic space, the resolution attained at the time of the study was insufficient to determine the exact location and structure of the protein (Motaleb et al., 2011).

Despite conflicting results for the functions of FliL in diverse species, it appears to help optimize motor performance to produce full torque in bacteria, especially in complex, viscous environments that require higher torque. This is reminiscent of the recently discovered gene *swrd*, which enhances the power of the flagellar motor under swarming conditions (Hall et al., 2018). FliL may therefore play a dual role in stabilizing the structure of the flagella and in optimizing stator function, thus enhancing bacterial motility.

## FliL Forms a Ring Structure Around the Stator Complex *in situ*

Like its function in flagellum-based motility, the structure of FliL had remained enigmatic until very recently. Takekawa et al. demonstrated in 2019 that the crystal structure of the C-terminal periplasmic region of FliL forms a 10-membered ring assembly within the crystal unit cell (Takekawa et al., 2019). Bioinformatic analyses showed that the FliL-ring is structurally homologous to proteins in the stomatin family, which form oligomeric complexes, including rings (Brand et al., 2012), to regulate ion channels. The size of the FliL-ring is compatible with that of the stator complex. In addition, mutational analyses suggest that FliL interacts directly with the stator units and rotor components, such as the MS-ring subunit FliF and C-ring protein FliG (Partridge et al., 2015; Zhu et al., 2015; Lin et al., 2018; Takekawa et al., 2019). Combining experimental evidence from diverse species, the authors proposed an intriguing model, whereby FliL forms a ring that surrounds the stator complex to regulate its ion channel activity, thus modulating torque (Takekawa et al., 2019; Zhou and Roujeinikova, 2021).

In two recent parallel studies (Guo et al., 2022; Tachiyama et al., 2022), cryo-ET and subtomogram averaging with focused refinement demonstrated that FliL indeed forms a ring-like structure around the stator units *in situ* in the flagellar motors of *B. burgdorferi* (Figures 2B–D,F,G) and of the stomach ulcer-causing human pathogen *Helicobacter pylori* (Tachiyama et al., 2022). Strikingly, in the absence of the stator complexes, FliL remains on the *B. burgdorferi* flagellar collar (a unique flagellar protein complex in spirochete motors) as a “half ring”-like structure (Figure 2F; Guo et al., 2022), indicating that the stator complex is required for proper assembly of FliL into a ring. This work further proposed that the FliL-half-ring “waits” for the stator complex to be recruited, then further multimerizes to encircle the stator complex and help stabilize its extended, active conformation, allowing ions to continuously flow through to generate torque.

To clarify the molecular basis for interactions between the FliL-ring and stator complex, structure prediction tools, such as the recently developed deep learning-based algorithm Alphafold2, were used to model the individual stator unit and FliL units within the *B. burgdorferi* motor (Jumper et al., 2021). These high-confidence molecular models are in excellent agreement with the ~20-Å cryo-ET map, revealing a pseudoatomic structure of the stator-FliL supramolecular complex (Figures 2C,D; Guo et al., 2022). The C-terminal periplasmic domain of FliL in *B. burgdorferi* (*Bb*FliL-ring) was modeled to oligomerize into a decameric ring in the same manner as that of the FliL-ring of *V. alginolyticus* resolved by X-ray crystallography. The *Bb*FliL-ring is situated above pentameric MotA embedded in the inner membrane (Figure 2C). While the flexible linker of MotB threads through the FliL-ring interior, MotB_PGB_ binds peptidoglycan above the ring (Figure 2C). The *Bb*FliL-ring would thus stabilize the stator complex in an extended, active conformation, whereby individual stator ion channels stay open for continuous ion flux. As 16 stator-FliL-supramolecular complexes can assemble around the *B. burgdorferi* motor, the *Bb*FliL-rings would influence the collective ion flow through the motor to help maximize torque.

Furthermore, the *Bb*FliL-ring provides MotB with a second anchoring point below MotB_PGB_ immobilized on the peptidoglycan (Figure 2C) and thus would help mitigate the torsional strain exerted on the MotB linker while MotA rotates rapidly beneath it. As the *Bb*FliL-ring in the periplasmic space encircles the MotA transmembrane ion channel, 10 N-terminal transmembrane domains of *Bb*FliL would form a fence-like structure to properly position the dynamic MotA within the inner membrane.

The *Bb*FliL-ring appears to act as a hub for extensive protein–protein interactions among motor components. Its ring exterior is embraced by various proteins that make up the spirochete-specific collar complex, while it interacts *via* multiple contact sites with the stator complex (Guo et al., 2022), providing an explanation for the low stator occupancy in the absence of FliL in *B. burgdorferi* (Figure 2E). FliL has relatively low shared sequence identity across species (Jenal et al., 1994; Takekawa et al., 2019). Though the oligomerization state and structure of FliL in other species are unknown, it is conceivable that FliL mediates an extensive network of protein-protein interactions that help stabilize the rotor and stator complexes. Further investigations are needed to test this hypothesis and to clarify the molecular basis for the observed phenotypes of bacteria, such as *E. coli* and *Salmonella*, which, in the absence of FliL, lose their flagellar filament under high-load conditions.

## The C-Ring Exhibits Remarkable Structural Plasticity During Rotational Switching

**Figure 3:**
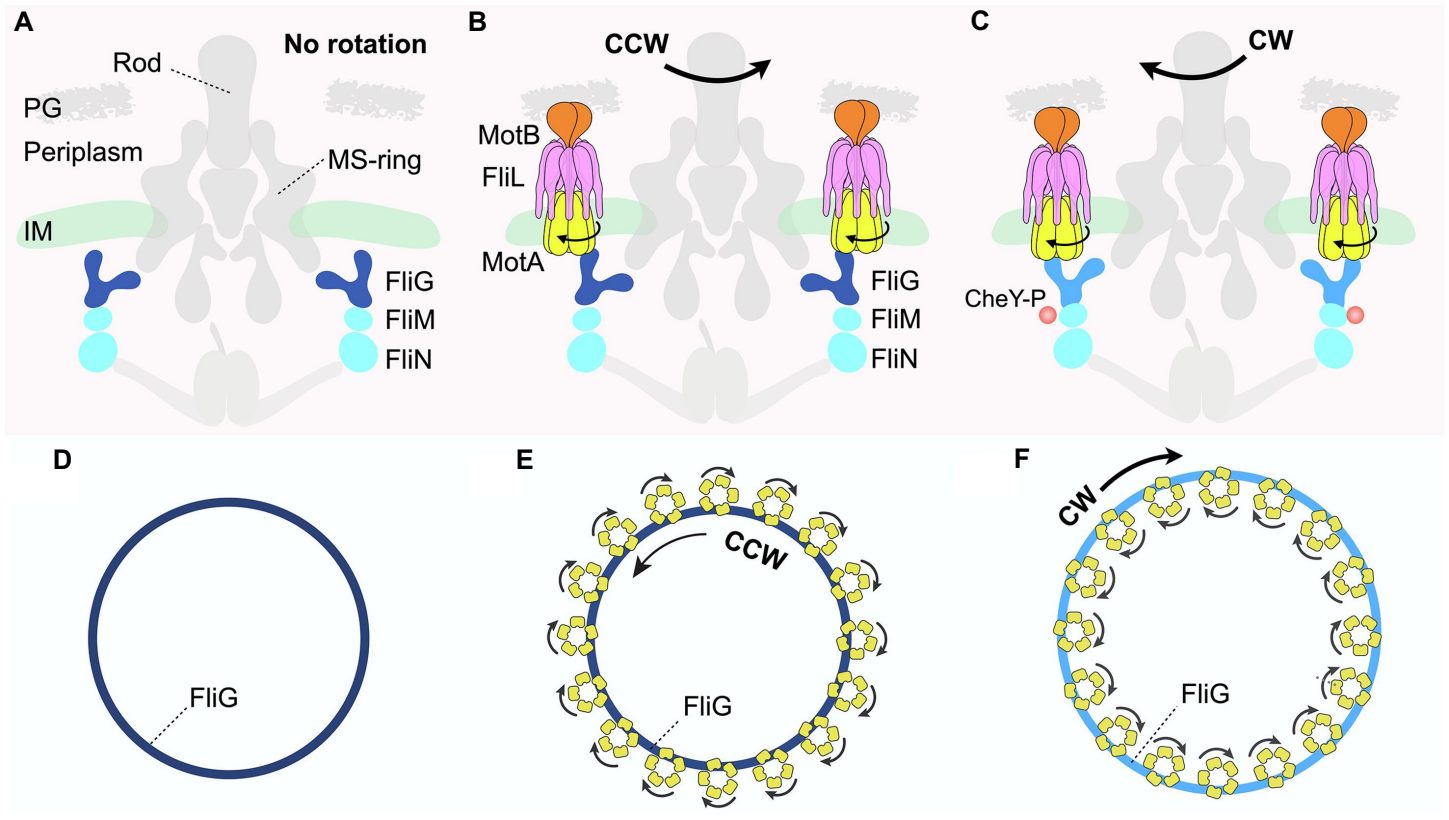
Distinct interactions between the C-ring and stator complexes in response to environmental signals regulate rotational switching of the flagellum. **(A,D)** Schematic diagrams depicting side and top views, respectively, of the *Borrelia burgdorferi* periplasmic motor in the absence of the stator complex. **(B,E)** Schematic diagrams illustrating the side and top views of the *B. burgdorferi* motor rotating in the CCW direction. The transmembrane FliL is colored pink in **(B)**. **(C,F)** Schematic diagrams illustrating the side and top views of the *B. burgdorferi* motor rotating in the CW direction when bound to a chemotactic protein, CheY-P. The FliG subunit is colored light blue in contrast to dark blue when rotating in the CCW direction.

All flagellated bacteria must be able to control their swimming direction *via* the bidirectional rotary motor to survive in various environments. The directional switch of the motor is regulated by the C-ring, which forms a cylindrical, wall-like structure at the base of the rotor in the cytoplasm (Liu et al., 2009; Wadhwa and Berg, 2021). Although protein components of each C-ring subunit are well conserved (Carroll and Liu, 2020), the oligomerization state and symmetry vary across species, resulting in significant differences in the size of C-rings and their capacity to generate torque. For instance, the canonical motor of *E. coli* has a relatively small C-ring with a ~34-fold symmetry. By contrast, the Lyme disease-causing spirochete bacterium *B. burgdorferi* has a significantly larger motor that powers the rotation of internal, periplasmic flagellar filaments (Tilly et al., 2008; Liu et al., 2009; Motaleb et al., 2011; Moon et al., 2016, 2018; Zhu et al., 2017; Chang et al., 2021), with a C-ring that has a 46-fold symmetry (Figures 3D–F). Due to this larger C-ring, 16 stator units can assemble around the motor of *B. burgdorferi*, whereas only ~12 stator complexes fit around that of *E. coli*. The higher number of power-generating stator units enables *B. burgdorferi* to produce higher torque and may be a key mechanism evolved by spirochetes to “drill” through viscous, mucus-like host environments that have high resistive forces.

Each C-ring subunit has a “Y-shaped” structure comprising three proteins: FliG, FliM, and FliN (Brown et al., 2005, 2007; Paul et al., 2011; McDowell et al., 2016; Figure 1 right panel). FliG comprises the upper arms of the “Y,” which interacts with the stator complex at one end while contacting the MS-ring subunit *via* the opposite arm (Figures 3A–C; Lee et al., 2010; Vartanian et al., 2012). FliM and FliN (Brown et al., 2005; Ahn et al., 2013; Notti et al., 2015), localized below FliG, interact with various effectors to help bacteria adjust motility in response to changing environments (Figures 1, 3A–C). These effectors include CheY and its analogs, which are well-characterized chemotactic signaling proteins that directly interact with the C-ring (Rossmann et al., 2020). One example discussed extensively in this review is the phosphorylated form of CheY, named CheY-P, which binds the C-ring to regulate flagellar rotational direction, thereby helping bacteria change swimming patterns in response to environmental signals. In the default state, the flagellar motor rotates in the counterclockwise (CCW) direction (Figures 3B,E). By contrast, the binding of CheY-P to FliM induces a conformational change in the C-ring subunit that causes the motor to switch and rotate in the clockwise (CW) direction (Dyer et al., 2009; Sarkar et al., 2010; Figures 3C,F). In addition, a recent study demonstrated that in *C. crescentus*, binding of the small molecule second messenger cyclic-di-GMP (c-di-GMP) induces conformational changes in a family of CheY-like proteins (Nesper et al., 2017). These CheY-like proteins in their c-di-GMP-bound states can then interact with the C-ring to regulate a range of motor activity, such as promoting the rapid attachment of motile bacteria to surfaces as seed for biofilm formation.

Although high-resolution structures of the individual C-ring subunits and CheY had been available, how multiple stator complexes interact with the intact C-ring *in situ* was unknown. The detailed mechanisms for motor rotation as well as directional switching had therefore remained unclear until recently. High-resolution structural biology tools, such as X-ray crystallography, nuclear magnetic resonance (NMR), and cryo-EM single-particle analysis, typically require proteins of interest to be isolated from their native environments within the organisms, an approach that often disrupts key protein–protein interactions necessary for the proper folding and assembly of complexes.

In recent years, cryo-ET, in conjunction with subtomogram averaging, has emerged as a leading technique to resolve the *in situ* structures of protein complexes within their native cellular context. In 2020, two parallel cryo-ET studies investigated the *in situ* structures of flagellar motors during rotation in both the CCW and CW directions in *V. alginolyticus* and *B. burgdorferi* (Carroll et al., 2020; Chang et al., 2020). Subtomogram averaging with focused refinement revealed that the C-rings in the flagellar motors of both bacteria undergo major remodeling upon CheY-P binding to switch the rotational direction. In the absence of the stator complexes, the intact C-ring of *B. burgdorferi* has a diameter of ~60 nm (Figures 3A,D). Upon stator association, the C-ring contracts slightly, rotating in the CCW direction (Figures 3B,E). Strikingly, binding of CheY-P to the base of the cylindrical, wall-like C-ring exterior causes it to expand into a larger structure that switches to rotate in the CW direction (Figures 3C,F). Critically, these large conformational changes of the C-ring alter how it interacts with the multiple stator units assembled around it (Figures 3B,C,E,F), providing a key to understanding the molecular mechanisms of both rotation and directional switching of the motor.

When the C-rings rotate in a CCW direction, the FliG subunits interact with the inner rims of the cone-shaped stator MotA subunit in the cytoplasm (Figures 3B,E). By contrast, upon binding of CheY-P to the C-ring subunits FliMN, FliG tilts outward to interact with the outer rims of MotA, resulting in the expansion of the C-ring (Figures 3C,F). Viewed from the top, 16 stator units surround and use their inner rims to contact the C-ring in the wild-type flagellar motor of *B. burgdorferi* (Figure 3E). As proton motive force (PMF) drives the rotation of the stator complexes in a CW direction, the C-ring would rotate in the CCW direction (Figure 3E). By contrast, CheY-P binding causes the C-ring to expand and interact with the outer rims of the stator complexes (Figures 3C,F). As the stator complexes rotate in the CW direction, they would then drive the C-ring to switch and rotate in the CW direction.

## New Insights Into Flagellar Motors as Sensors for Various Environments

In addition to propelling motility, the flagellar motor is a key component in sensing mechanical and chemical stimuli, thus helping bacteria adapt to various environments (Hug et al., 2017; Hughes and Berg, 2017). As described above, the C-ring undergoes large structural remodeling upon receiving chemotactic signals from the environment to regulate motor activity, such as switching rotational direction. Mechanosensing by flagellar motors is intimately associated with the change in total ion flux through the stator units (Lele et al., 2013; Belas, 2014; Chawla et al., 2017; Nord et al., 2017; Laganenka et al., 2020; Antani et al., 2021; Wadhwa et al., 2021), which interact with the rotor in a dynamic fashion in response to external load changes (Figures 2E–G; Nirody et al., 2016; Nord et al., 2017; Sato et al., 2019; Nakamura et al., 2020). The stators units dissociate from the motor more quickly in low-load conditions than in high-load environments (Wadhwa et al., 2019). Given the observed intimate interactions between the FliL-ring and the active stator units, this raises the question: does FliL play a role in the regulation of mechanosensing by the flagellar motor?

The assembly of the FliL-half-ring into a full ring may provide insight into this key question. Cryo-ET studies with *B. burgdorferi* reveal that even before the diffusive stator complexes are recruited, FliL has oligomerized into a half ring (Figure 2F). Once a stator complex contacts the rotor, its interaction with the C-ring and the PMF would induce remodeling of the stator complex from an inactive, compact form to an extended, active conformation to enable passage of ions through the transmembrane channel. As loss of FliL does not abolish motor rotation in many bacteria, including *B. burgdorferi* (Raha et al., 1994; Attmannspacher et al., 2008; Motaleb et al., 2011; Partridge et al., 2015; Takekawa et al., 2019), activation of the stator complex is independent of the presence of FliL. Nevertheless, once the stator complex is activated near the FliL-half-ring, FliL would continue to oligomerize into a full ring to help stabilize the stator complex in the extended conformation (Figures 2C,G). Under low-load conditions, as the stator units have high dissociation rates, many of them might detach from the FliL-half-rings before the full rings can form. We speculate that it is possible that some of these stator units are in their inactive conformation to limit ion flux, and their compact shape cannot fit within the narrow constricting interior of the FliL-rings to help better stabilize them around the rotor. Indeed, we observed that the PMF-blocking D24N mutation of MotB not only causes the reduction of *B. burgdorferi* stator occupancy by over 50% (Chang et al., 2019) but also significantly disrupts the FliL-ring formation. By contrast, the stator complex dissociates more slowly in high-load conditions. The longer retention time of the stator complex should allow adequate time for the FliL-half-ring to oligomerize and further stabilize the unplugged, active stator complex within a full ring to help maximize ion flux and generate high torque (Figures 2F,G). We speculate that the FliL-ring-stator association is not permanent, given that excessive ion leakage is harmful to bacteria. However, the kinetics for stator dissociation would be slower as the FliL-ring needs to disassemble first before clearing a path for the stator complex to diffuse away from the motor (Figures 2F,G). In particular, the dissociation rate of the stator units might be even smaller for spirochete motors, whose collar structures further envelop the stator-FliL-supramolecular complexes (Chang et al., 2021; Guo et al., 2022). The presence of accessory scaffold proteins around the stator units may be an adaptation mechanism evolved by spirochetes to sustain motility in order to survive and cause disease in various complex host environments (Zhou and Roujeinikova, 2021).

Unique aspects of the stator-FliL-supramolecular complex may very well have enabled its discovery and structural elucidation because spirochetes have evolved an intricate, intrinsically high-duty-ratio motor stabilized by the collar. Similarly, although the *H. pylori* motor lacks a collar, it contains a different periplasmic scaffold, referred to as the “cage” (Qin et al., 2017; Zhou and Roujeinikova, 2021), which is also necessary for stabilizing stator-FliL-supramolecular complexes to generate full torque. This may be why the stator-FliL-supramolecular complex in *H. pylori* can be observed by cryo-ET, as in *B. burgdorferi*. For bacteria, such as *E. coli*, which naturally reside in less complex liquid environments with lower external load, the stator units are likely too dynamic to be captured in association with the rotor. It would thus be intriguing to test whether stator-FliL-supramolecular complexes are sufficiently stable to be seen by cryo-ET and subtomogram averaging *in situ* when bacteria with these dynamic motors are subjected to higher external loads.

## Conclusion and Future Perspectives

Remarkable technical breakthroughs in cryo-EM over the past few years have laid a solid foundation for answering fundamental questions: how do flagellar motors generate torque mediated by ion motive force, and what is the structural basis for switching rotational direction? Combining a wealth of knowledge of the biophysics, biochemistry, and genetics of rotary motors with an integrative structural biology approach that includes increasingly powerful machine learning-based structure prediction methods (e.g., Alphafold2) will elucidate the mechanisms of the flagellar motor in response to various environments. One exciting challenge is to directly visualize stator units in association with the rotor in diverse bacteria *via in situ* methods, such as cryo-ET. This approach stands not only to validate the proposed model to unravel the structure and function of FliL in various species but also to open opportunities to delve into species-specific flagellar features (e.g., the collar in spirochetes) required by bacteria to generate full torque across different environments. In addition, revealing the structural basis for how the C-ring and stator units remodel in response to binding by a range of chemotactic molecules, such as CheY analogs, will yield mechanistic explanations for the fascinating question of how bacteria use the flagellar motor to accelerate, brake, and initiate colonization in distinct niches in response to environmental cues. Such future studies hold great promise for researchers to design inhibitors of motility in pathogens and to engineer more powerful motors in synthetic microorganisms for wide-ranging clinical and biomedical applications.

## Author Contributions

SG and JL wrote the manuscript and approved it for publication.

## Funding

SG and JL are supported by grants R01AI087946 and R01AI132818 from National Institute of Allergy and Infectious Diseases (NIAID) and National Institutes of Health (NIH). SG is also supported by a CIHR fellowship from the Canadian Institutes of Health Research.

## Conflict of Interest

The authors declare that the research was conducted in the absence of any commercial or financial relationships that could be construed as a potential conflict of interest.

## Publisher’s Note

All claims expressed in this article are solely those of the authors and do not necessarily represent those of their affiliated organizations, or those of the publisher, the editors and the reviewers. Any product that may be evaluated in this article, or claim that may be made by its manufacturer, is not guaranteed or endorsed by the publisher.
